# From mechanism to therapy: the journey of CD24 in cancer

**DOI:** 10.3389/fimmu.2024.1401528

**Published:** 2024-05-31

**Authors:** Kai Zhao, Caifeng Wu, Xiangjun Li, Mengchao Niu, Dan Wu, Xiaofeng Cui, Hai Zhao

**Affiliations:** ^1^ Department of Neurosurgery, The Affiliated Hospital of Qingdao University, Qingdao, China; ^2^ Department of Hand and Foot, The Affiliated Hospital of Qingdao University, Qingdao, China; ^3^ Department of Breast Surgery, The Affiliated Hospital of Qingdao University, Qingdao, China; ^4^ Department of Operation Room, The Affiliated Hospital of Qingdao University, Qingdao, China

**Keywords:** CD24, innate checkpoint, phagocytosis, therapeutic target, cancer

## Abstract

CD24 is a glycosylphosphatidylinositol-anchored protein that is expressed in a wide range of tissues and cell types. It is involved in a variety of physiological and pathological processes, including cell adhesion, migration, differentiation, and apoptosis. Additionally, CD24 has been studied extensively in the context of cancer, where it has been found to play a role in tumor growth, invasion, and metastasis. In recent years, there has been growing interest in CD24 as a potential therapeutic target for cancer treatment. This review summarizes the current knowledge of CD24, including its structure, function, and its role in cancer. Finally, we provide insights into potential clinical application of CD24 and discuss possible approaches for the development of targeted cancer therapies.

## Introduction

Generally, cancer cells are typically eliminated by the intricate mechanisms within the human immune system. However, they can develop resistance to the body’s anti-tumor immune responses, allowing them to evade immune surveillance (1). Cancer immunotherapy has transformed the field of oncology by harnessing the patient’s immune system to combat cancer cells. This can be accomplished through two main approaches: immune checkpoint-targeted therapy and the transfer of modified immune cells. Both methods involve manipulating the immune system to identify and attack cancer cells. Immune checkpoint inhibitors, such as antibodies targeting programmed cell death ligand 1 (PD-L1) or cytotoxic T-lymphocyte-associated protein 4 (CTLA-4), as well as stimulants of other co-stimulatory molecules that override inhibitory pathways to enhance immune function, have shown success in several clinical trials (2–4). However, they still encounter challenges like low response rates, high expenses, and non-specific toxicity (5). The adoptive transfer of cells primarily involves genetically modified cells, such as chimeric antigen receptor (CAR)-T cells, and various other cell types (6, 7). Cancer immunotherapy has seen significant progress with the clinical success of immune checkpoint blockade and CAR-T-cell therapies in recent years. It has emerged as an innovative and potent clinical approach, offering unique advantages over traditional anti-tumor treatments like surgery, radiotherapy, and chemotherapy.

Most of the immunotherapies developed in the past primarily aimed at stimulating adaptive immunity, especially by reinvigorating and enhancing T cell responses. However, recent research has revealed that innate immune checkpoints expressed on antigen-presenting cells (APCs) play a pivotal role in immune evasion (8–10). Some checkpoints are responsible for detecting and eliminating cancer cells through phagocytosis while also restraining the innate immune response (11, 12). Cancer cells use various mechanisms to evade macrophage-mediated clearance. One of these mechanisms involves the upregulation of specific anti-phagocytic membrane proteins often referred to as “don’t eat me” signals. These proteins include cluster of differentiation 47 (CD47) (13), cluster of differentiation 24 (CD24) (14, 15), PD-L1 (16), the beta-2 microglobulin (β2M) subunit of the major histocompatibility class I complex (MHC-I) (17), stanniocalcin 1 (STC-1) (18), and GD2 (19). Phagocytosis is typically assisted by inherent “eat me” signals that serve as ligands for phagocytic receptors. These signals can initiate significant changes in the cytoskeleton, leading to the engulfing of the target.

**Figure 1:**
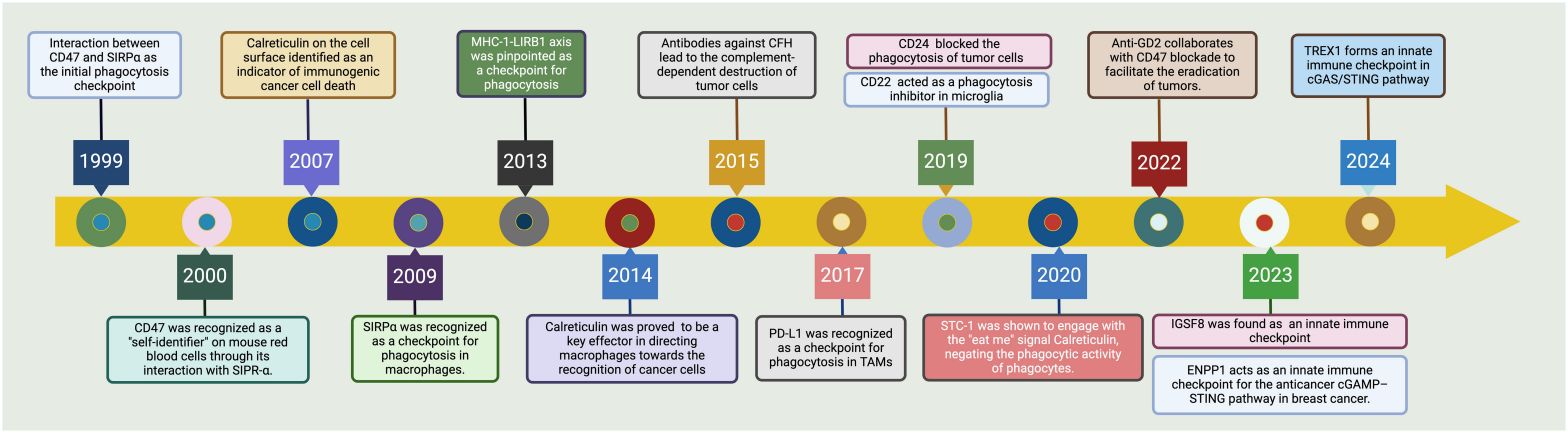
Tracing the advancements in phagocytosis checkpoints for cancer. The CD47-SIRPα axis emerged as the seminal checkpoint axis in 1999, marking the entry into phagocytosis checkpoint exploration (22, 23). By 2007, cell surface calreticulin had been recognized as a decisive marker signaling immunogenic cell death in cancers (24). The upregulation of CD47 on malignancies was noted in 2009, setting a precedent for targeted therapies aiming to boost phagocytosis (25, 26). Following these discoveries, by 2013, the MHC-I-LIRB1 interaction was pinpointed as another checkpoint avenue (27). In a significant turn of events in 2015, antibodies against Complement Factor H (CFH) were found to lead to the complement-dependent destruction of tumor cells (28). This was followed by the identification of PD-L1 as a checkpoint for phagocytosis in tumor-associated macrophages (TAMs) in 2017 (29). By 2019, the field had recognized CD24 and CD22 as additional modulators of phagocytosis, with CD24 impeding and CD22 promoting this critical immune process within microglia (30). The latest key findings in 2022 showed the interaction of STC-1 with calreticulin, attenuating the phagocytic activity of immune cells (18). IGSF8 has been identified as a new target for cancer immunotherapy due to its role in regulating the innate immune system in 2023 (31). Consequently, a phase 1 clinical trial has started to test the IGSF8 inhibitor GV20-0251 in individuals with advanced or metastatic solid tumors, under the study number NCT05669430 (32). In the latest advancement by 2024, the TREX1 enzyme has been identified as a limiting factor for the cGAS/STING-mediated antitumor immunity, presenting new therapeutic windows (33). This historical trajectory underscores the rapid evolution and complex interplay of innate immune mechanisms that are currently shaping novel cancer immunotherapeutic strategies. *Created with BioRender.com*.

In recent times, the focus on phagocytosis checkpoints, notably CD24, has increased as potential targets for treating cancer and non-neoplastic conditions (20, 21) (**Figure 1**). CD24 has shown elevated levels in several cancers like breast, prostate, pancreatic, and ovarian cancers. Due to its diverse post-translational modifications, CD24 has been implicated in tumor development, invasion, and metastasis. It is also considered as a potential marker for cancer prognosis and therapy (34). Moreover, emerging studies have unveiled the crucial roles of CD24 in various health conditions, including autoimmune diseases, sepsis metabolic disorders, and graft vs host diseases (35–39).

CD24 is a glycosylphosphatidylinositol-anchored protein known as a B-cell differentiation antigen in 1978 (40). It is present in various tissues and cell categories, such as hematopoietic stem cells, B and T lymphocytes, epithelial cells, and neural cells (34, 41). CD24 has also been found to play a role in a variety of physiological and pathological processes, including cell adhesion, migration, differentiation, and apoptosis (42). In this review, we provide an overview of CD24 biology from fundamental concepts, paired receptors and relevant signal pathways, to the possibility of targeting CD24 as a novel therapeutic target. Then we highlight the ongoing clinical advancements in the targeting CD24 and identify the challenges and potential solutions in the context of cancer immunotherapy. Our aim is that this comprehensive review will not only enhance our understanding of the current state of research on CD24 but also explore the potential of CD24-based immunotherapy.

## Structure of CD24

CD24 is a small glycosylphosphatidylinositol-anchored protein that is composed of a short extracellular domain, a single transmembrane domain, and a cytoplasmic tail (43). The extracellular domain of CD24 contains a variable number of N-linked and O-linked glycosylation sites, which are involved in the regulation of CD24-mediated cell adhesion and signaling (44). The CD24 protein is encoded by the CD24 gene located on chromosome 6p21.3, which consists of 80 amino acids and has a molecular weight of approximately 27 kDa (45) It contains a single N-glycosylation site and several O-glycosylation sites, which contribute to its glycosylation status. The protein is anchored to the cell membrane via a glycosylphosphatidylinositol (GPI) anchor at its C-terminus (44). The crystal structure of CD24 has been determined by X-ray crystallography. It forms a compact, globular structure with a β-barrel fold composed of 4 antiparallel β-strands. The β-barrel is stabilized by a disulfide bond between Cys53 and Cys73. The N-terminal part of the protein contains a short α-helix and a flexible loop region (46).

The extracellular domain of CD24 is highly glycosylated and forms a distinct patch on the surface of the protein (44). The glycosylation of CD24 has been shown to be important for its function, as it mediates interactions with other proteins and can modulate its biological activity (21). This process is integral to the role of CD24 in cell adhesion and signaling due to its highly glycosylated extracellular domain (44). This domain contains N-linked and O-linked glycosylation sites which are crucial for the interaction with cell surface receptors like P-selectin, Siglecs, and β1 integrin, which are involved in cell adhesion, migration, and immune response regulation​​ (47, 48).

The activity of CD24 is influenced by interactions with various proteins across different cellular contexts. Notably, CD24 binds to P-selectin on platelets and endothelial cells, facilitating tumor cell migration and metastasis. CD24 also interacts with Siglec-10 on immune cells, dampening immune responses and contributing to immune evasion in tumors (49). Additionally, CD24 activates Src Family Kinases, triggering signaling pathways that support cell proliferation, survival, and migration (50). Furthermore, its association with integrins affects cell adhesion, migration, and invasion, underscoring its role in cancer dynamics (51). The functional outcomes of CD24 interactions are contingent on the cellular environment and the intricate balance between its binding partners and the resultant signaling cascades.

## A brief overview of phagocytosis checkpoints beyond CD24

**Figure 2:**
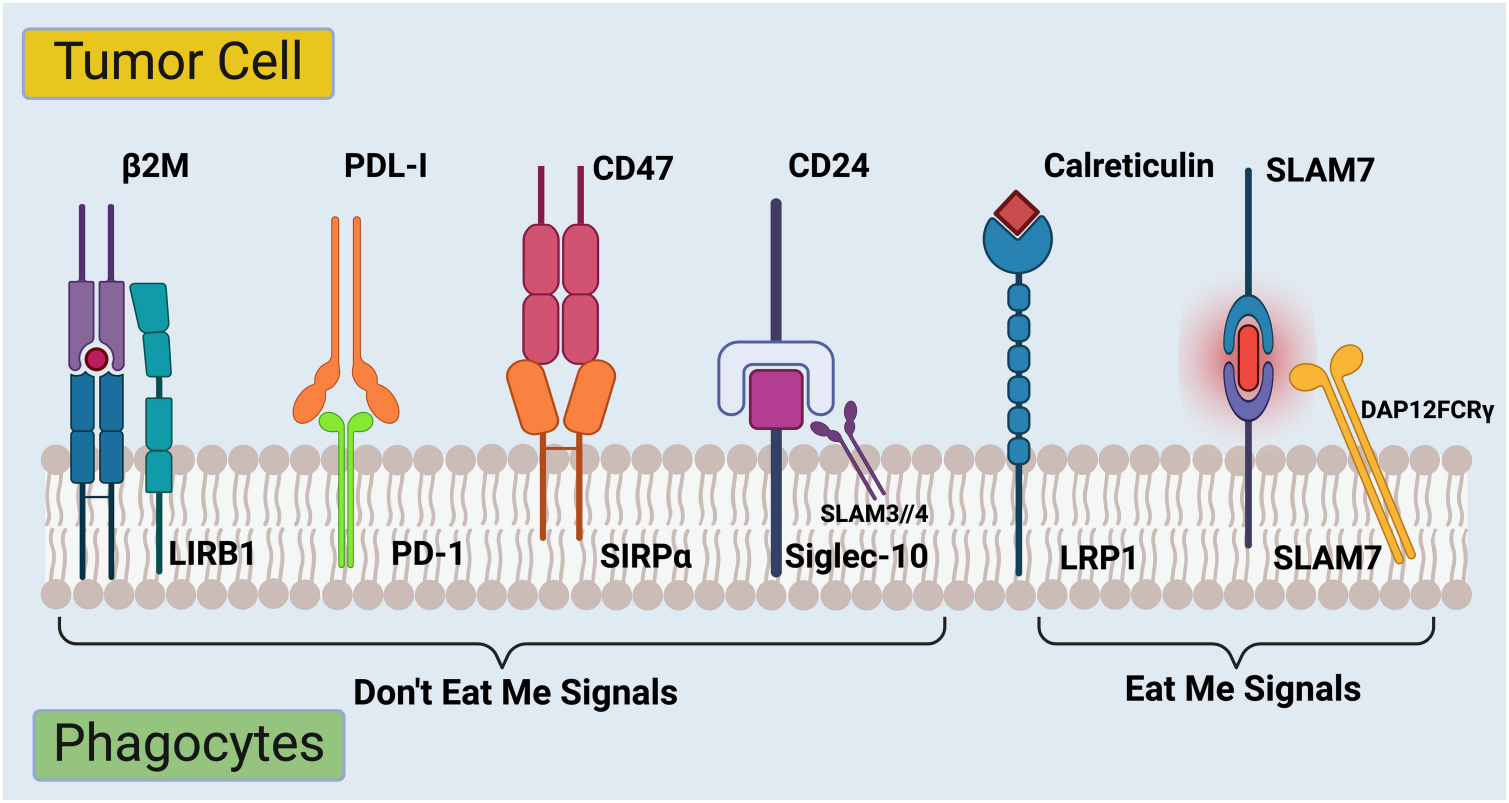
Regulation of phagocytosis in cancer immunotherapy checkpoints. The complex interplay between signals that regulate the ability of immune system to recognize and destroy cancer cells. On the left side are molecules labeled “Don’t Eat Me Signals.” These are proteins expressed on the surface of tumor cells that interact with receptors on immune cells called phagocytes. The interaction inhibits the phagocytes from engulfing and destroying the tumor cells. Firstly, β2M (Beta-2 microglobulin) is part of the MHC class I complex and can interact with LIRB1 (Leukocyte immunoglobulin-like receptor B1) on phagocytes (53). Secondly, PDL-1 (Programmed death-ligand 1) binds to PD-1 (Programmed death-1) on immune cells, delivering an inhibitory signal to the immune cells (54). Thirdly, upon binding to SIRPα (Signal-regulatory protein alpha) on phagocytes, CD47 transmits a signal to the phagocyte to not ingest the cell (55). Lastly, CD24 binds to Siglec-10 (Sialic acid-binding Ig-like lectin 10) on immune cells, which can also prevent phagocytosis (56). On the right side of the image, there are several “Eat Me Signals.” Calreticulin, when exposed on the surface of stressed or dying cells, interacts with the LRP1(low-density lipoprotein receptor-related protein 1) on phagocytes and prompts the immune cells to engulf those cells (57, 58). SLAM7 (Signaling lymphocytic activation molecule 7) pairs with DAP12FCRγ (DNAX activation protein 12 Fc receptor gamma), which is a signaling adaptor for natural killer cells, enhancing the immune response against the tumor cell (59). The balance between these signals determines whether the immune system will recognize and attack the tumor cell or not. Targeting these pathways is a promising strategy for cancer immunotherapy, aiming to tip the balance towards “Eat Me Signals” to promote the removal of cancer cells by the immune system. *Created with BioRender.com*.

Ever since the CD47-Signal-Regulatory Protein α (SIRPα) axis was identified as the initial checkpoint for tumor phagocytosis in the late 2000s, several other phagocytosis checkpoints responsible for enabling tumor cells to evade phagocytic elimination have come to light (12). These include CD24-Siglec 10 interaction, PD-1-PD-L1 and LILRB1-β2M interaction (52) (**Figure 2**).

### CD47-SIRPα interaction

The CD47-SIRPα pathway is the most extensively researched checkpoint for phagocytosis in cancer (9, 60, 61). Treatments aimed at blocking the interaction between CD47 and SIRPα are the most advanced in clinical trials and are currently being explored in the clinic for various human cancers (62–65).

CD47-SIRPα interaction is a biological process in which the protein CD47 on the surface of various cells, including cancer cells, interacts with SIRPα on the surface of macrophages. This interaction sends a “don’t eat me” signal to macrophages, preventing them from engulfing and destroying the CD47-presenting cells. This mechanism is often exploited by cancer cells to evade the immune system and avoid phagocytosis, allowing them to survive and proliferate. It has been a focus of cancer immunotherapy research to develop strategies that disrupt or block this interaction, enabling the immune system to recognize and eliminate cancer cells more effectively (**Figure 2**).

The earlier clinical application of the SIRPα-CD47 interaction in cancer therapy has shown promise, primarily utilizing anti-CD47 monoclonal antibodies to block the CD47 “don’t eat me” signal. However, in recent developments, Gilead Sciences, Inc. faced setbacks with its immunotherapy drug, Magrolimab, in clinical trials. The Phase 3 ENHANCE-3 study, which aimed to treat newly diagnosed acute myeloid leukemia patients with Magrolimab in combination with venetoclax and azacitidine, was discontinued due to futility in improving overall survival and increased risk of death, primarily from infections and respiratory failure. Consequently, FDA placed a full clinical hold on all Magrolimab studies in AML and MDS, including related expanded access programs. These challenges with Magrolimab have intensified interest in alternative therapeutic targets such as CD24. More and more research are now increasingly focusing on the potential of CD24 as a novel target in cancer treatment, spurred by the need to find more effective and safer treatment options in light of the setbacks experienced with CD47-targeted therapies. This shift underscores the ongoing search for better cancer treatments and the importance of exploring diverse therapeutic targets.

### MHC-I–LILRB1 axis

The MHC-I-LILRB1 axis is an immune regulatory pathway involving Major Histocompatibility Complex Class I (MHC-I) and Leukocyte Immunoglobulin-Like Receptor 1 (LILRB1) (17, 66). LILRB1 transmits inhibitory signals that can reduce the activation and phagocytic activity of these cells. This mechanism is particularly relevant in the tumor microenvironment where cancer cells exploit this pathway to escape immune-mediated destruction (66). By presenting MHC-I molecules that engage LILRB1, cancer cells can inhibit the phagocytic function of immune cells, thereby evading immune surveillance. In cancer immunotherapy, blocking the interaction between MHC-I and LILRB1 is considered a promising strategy to enhance the immune system to recognize and destroy cancer cells, thereby facilitating more effective phagocytosis and elimination of malignant cells (17).This interaction can send inhibitory signals that dampen immune responses, preventing the immune system from attacking cells displaying MHC-I, which can include cancer cells. In cancer immunotherapy, strategies aim to block this interaction, allowing the immune system to better recognize and target cancer cells for destruction (52, 67).

### PD-1–PD-L1 axis

The PD-1-PD-L1 axis is a crucial immune checkpoint pathway that significantly impacts the immune system to respond to cancerous and pathogenic cells (68). PD-1 is a receptor found on T cells, while PD-L1 is expressed on various cells, including many cancer cells (69, 70). The interaction between PD-1 and PD-L1 sends an inhibitory signal that reduces T cell activity and dampens the overall immune response (71). Crucially, recent studies have shown that PD-L1 is also involved in regulating the phagocytic functions of macrophages and dendritic cells (72, 73). By engaging with PD-1 on these cells, PD-L1 can decrease their phagocytic activity, further aiding cancer cells in evading immune detection and destruction (29, 72, 73). In cancer immunotherapy, targeting this axis with PD-1 or PD-L1 inhibitors enables a more robust immune attack by not only enhancing T cell activity but also by potentially increasing the phagocytic capabilities of macrophages and dendritic cells, allowing for more effective recognition and elimination of tumor cells (54, 74).

### STC-1

STC-1, or Stanniocalcin-1, is a glycoprotein that plays a nuanced role in the regulation of cellular calcium and phosphate homeostasis under physiological conditions (75–77). Beyond its traditional functions, recent research has highlighted its emerging role as an immune checkpoint in cancer biology (78–80). STC-1 is implicated in modulating the tumor microenvironment, particularly influencing the behavior of immune cells (81). It contributes to the suppression of immune responses by inhibiting macrophage activation, which in turn can promote tumor growth and metastasis by allowing cancer cells to evade immune detection and destruction (82–84).

In the context of cancer, STC-1 can be viewed as a potential therapeutic target. Its ability to modulate immune responses presents an opportunity for novel cancer therapies aimed at enhancing the ability of immune system to recognize and eliminate tumor cells (18, 85–87). Targeting STC-1 could potentially disrupt its immunosuppressive effects, thereby enhancing the efficacy of existing immunotherapeutic approaches such as checkpoint inhibitors or CAR-T cell therapies (88). However, the specific mechanisms through which STC-1 interacts with other components of the immune system and its broader implications for cancer therapy are still under investigation. Further research is required to fully understand its role and to harness its potential in oncology.

### CD22

CD22 is one of Siglec (sialic acid-binding immunoglobulin-type lectin) that primarily functions as an immune checkpoint on B cells (89). It is expressed on the surface of mature B cells and to a lesser extent on some early B cells, playing a critical role in modulating B cell signaling and activation (90, 91). As an immune checkpoint, CD22 helps to maintain immune tolerance and prevent autoimmune responses by regulating the threshold for B cell receptor (BCR) signaling (92).

In the context of cancer, especially in certain types of B cell leukemias and lymphomas, CD22 is of particular interest because it can be overly expressed (93). This overexpression can contribute to the survival and proliferation of malignant B cells. Targeting CD22, therefore, presents a promising strategy for cancer immunotherapy (94). By inhibiting or modifying the function of CD22 through monoclonal antibodies or other biologic agents, it is possible to enhance the ability of immune system to attack cancerous B cells. Such strategies can potentially disrupt the protective signals that CD22 provides to tumor cells, thereby enhancing the efficacy of treatments aimed at eradicating B cell malignancies (95, 96).

Additionally, the role of CD22 in immune suppression and its selective expression on B cells makes it an attractive target for selective therapies that aim to minimize off-target effects and maximize the immune response against the tumor cells. This approach is still under investigation, with ongoing research focusing on how best to leverage CD22 targeting for therapeutic benefits in hematological cancers (7).

### GD2

GD2 was discovered as a neuroblastoma tumor antigen in the 1980s (97). The expression of GD2 in normal tissues is limited, primarily detected in the brain, spinal cord, and skin melanocytes (98). It is thought to play a role in neural differentiation and repair, yet further research is needed to elucidate the specific underlying mechanisms (98, 99). For example, mice lacking GM2/GD2 synthase (B4GALNT1) show reduced myelination in the central nervous system, demyelination in peripheral nerves, and axonal degeneration (100). These mice then develop progressive behavioral neuropathies, which demonstrates the importance of GM2/GD2 in maintaining normal neural functions (101). While the precise function of GD2 in normal cell physiology remains to be fully understood, it is known to enhance cancer cell proliferation, adhesion, migration, invasion, and resistance to apoptosis.

As a disialoganglioside, GD2 is notably overexpressed in neuroblastoma, sarcomas, gliomas, and neuroendocrine tumors (97, 102, 103). The significance of GD2 as a cancer target has been extensively reviewed (103, 104). GD2 antibodies lies in their ability to bind selectively to the GD2 antigen on cancer cells, thereby facilitating the immune system recognition and destruction of these cells. This targeted approach aims to minimize damage to normal tissues, given the restricted expression of GD2 in the body. The clinical application of anti-GD2 antibodies represents a significant advancement in the treatment of certain types of cancer, particularly neuroblastoma (105). Clinical trials have demonstrated that anti-GD2 therapy can significantly prolong the survival of patients with high-risk neuroblastoma (105). The mechanism of action involves antibody-dependent cellular cytotoxicity (ADCC), where the binding of anti-GD2 antibodies to GD2-expressing tumor cells triggers their elimination by natural killer (NK) cells, macrophages, and other immune effector cells (106). Moreover, anti-GD2 antibodies have been explored in combination with other therapeutic modalities, such as chemotherapy, immunomodulators, and radiotherapy, to enhance their anti-tumor efficacy (107, 108). In summary, anti-GD2 antibodies represent another promising therapeutic strategy for targeting GD2-expressing tumors. Ongoing research and clinical trials continue to refine and expand their application, aiming to maximize their therapeutic potential while minimizing adverse effects.

### ENPP1

ENPP1 (Ectonucleotide Pyrophosphatase/Phosphodiesterase) is increasingly recognized as an important immune checkpoint in cancer immunobiology, playing a critical role in the modulation of the tumor microenvironment and immune evasion (109, 110). ENPP1 is involved in the hydrolysis of ATP and GTP to AMP and GMP, thereby influencing the levels of these molecules in the extracellular environment (111–113). In the context of cancer, ENPP1 overexpression is associated with the development of an immunosuppressive TME (110, 114). This occurs through the modulation of adenosine levels, which can suppress immune responses and facilitate tumor progression.

ENPP1 impacts cancer immunity primarily by shifting the ATP-adenosine balance towards adenosine, an immunosuppressive agent that inhibits effector T cells and promotes regulatory T cell activities (115, 116). This shift is crucial for cancer cells to evade immune surveillance. Moreover, ENPP1 intersects significantly with the cGAS-STING pathway (117). By degrading cGAMP, a secondary messenger in the STING pathway, ENPP1 dampens the immune response to cancer cells (118, 119).

The inhibition of ENPP1 presents a promising avenue for cancer therapy. Blocking ENPP1 can potentially restore the effectiveness of the STING pathway, thereby enhancing immune-mediated tumor suppression. This can be particularly effective when used in combination with other therapies such as checkpoint inhibitors, radiation therapy, or DNA damage response inhibitors, which could synergize to amplify the anti-tumor immune response (120, 121).

Various small molecule inhibitors targeting ENPP1 have shown efficacy in preclinical models, suggesting potential for clinical applications. These inhibitors not only counteract the immunosuppressive effects of ENPP1 but also may enhance the effects of other immunotherapeutic strategies by enabling a more robust immune response against tumors (121–123).

In conclusion, targeting ENPP1 offers a dual benefit in cancer therapy by potentially disrupting the immunosuppressive adenosine pathway and by reactivating important immune surveillance mechanisms like the STING pathway. This dual action makes ENPP1 a compelling target in the landscape of cancer immunotherapy.

### IGSF8

IGSF8 (Immunoglobulin superfamily member 8, also known as CD316 or EWI-2) plays a pivotal role as an innate immune checkpoint in cancer immunology (31, 124). This molecule is primarily expressed on cell membranes and exerts its effects through unique transmembrane interactions (125–127). Recent studies have unveiled that IGSF8 is highly expressed in several cancer types, where it significantly modulates the immune microenvironment by interacting with key immune cells like natural killer (NK) cells and dendritic cells (DCs) (31, 125, 128).

IGSF8 mechanism involves suppressing NK cell cytotoxicity and impeding the antigen presentation capabilities of dendritic cells, which collectively contribute to immune evasion by tumors (31). This suppression is crucial in cancers with low antigen presentation and poor immune cell infiltration, correlating with advanced disease stages and poorer patient prognoses.

Clinical relevance is underlined by the development of antibodies targeting IGSF8, such as GV20-0251 (32). This antibody has shown promising results in preclinical models, enhancing NK cell-mediated cytotoxicity and improving antigen presentation (31). The therapeutic potential of IGSF8 targeting is further highlighted in ongoing clinical trials aimed at evaluating the efficacy of IGSF8 inhibitors in treating advanced or metastatic solid tumors. These findings position IGSF8 not only as a marker of immune evasion but also as a viable target for novel immunotherapeutic strategies in cancer treatment​​​​​​.

### TREX1

TREX1 (Three prime repair exonuclease 1) acts as an innate immune checkpoint, particularly affecting the cGAS-STING pathway which is critical in the immune response against cancer (33). The role of TREX1 is to degrade cytoplasmic DNA, thereby preventing the activation of the cGAS-STING pathway (129). This pathway is involved in detecting cytoplasmic DNA and initiating an immune response through the production of type I interferons, which are crucial for immune surveillance against tumors (130).

In the absence of TREX1, accumulation of cytoplasmic DNA would typically lead to increased activation of the cGAS-STING pathway, enhancing immune responses (131–133). However, in cancer cells, TREX1 helps tumors evade this immune surveillance (129, 134). High levels of TREX1 in tumor cells can lead to decreased activation of the cGAS-STING pathway, allowing cancer cells to avoid detection and destruction by the immune system. Understanding the function of TREX1 as an immune checkpoint has implications for cancer therapy. Targeting TREX1 might enhance the effectiveness of immunotherapies by increasing the immune system’s ability to recognize and destroy tumor cells through the activation of the cGAS-STING pathway (135–138).

Research is ongoing to explore the potential of targeting TREX1 in cancer treatment, aiming to counteract its immunosuppressive effects and improve the efficacy of cancer immunotherapies.

## Normal physiological roles and significance of CD24

In the realm of cellular biology, CD24 emerges as a pivotal GPI-anchored cell surface protein, manifesting an expansive expression across diverse tissues and cell types. Recognized initially as a marker of B-cell differentiation, the scope of CD24 function extends far beyond, encompassing roles in critical physiological processes such as cell adhesion, migration, differentiation, and apoptosis. The highly glycosylated extracellular domain of CD24 facilitates its interaction with a multitude of cell surface receptors including P-selectin, Siglecs, and β1 integrin, thereby playing a vital role in mediating cell-cell communication, immune response regulation, and tissue homeostasis (139). The functional modulation of CD24 through its glycosylation status underscores its capacity to influence biological activity significantly. For instance, the interaction of CD24 with P-selectin not only promotes tumor cell migration and metastasis but also reveals a mechanism for immune evasion by engaging Siglec-10 on immune cells, thus dampening immune responses (140, 141). Furthermore, the activation of Src Family Kinases by CD24 triggers signaling pathways that are instrumental in supporting cell proliferation, survival, and migration (142, 143). This overview of CD24 under normal physiological conditions elucidates the multifaceted roles it plays, highlighting the importance of understanding these fundamental processes to grasp the implications of CD24 dysregulation in disease states, notably in cancer.

## CD24 as a novel phagocytosis checkpoint

CD24 is emerging as a pivotal modulator in the immune evasion mechanisms of cancer cells, acting as a novel phagocytosis checkpoint that manipulates the immune system to recognize and eliminate malignant cells (8, 56, 144). Through its interaction with the inhibitory receptor Siglec-10 on macrophages, CD24 effectively sends a “don’t eat me” signal, thereby preventing the phagocytosis of cancer cells (49). This interaction not only shields cancer cells from immune-mediated destruction but also contributes to the complexity of tumor-immune dynamics, complicating therapeutic interventions aimed at enhancing immune responses against tumors (56).

Recent studies elucidate the molecular pathways underpinning the CD24-Siglec-10 axis, highlighting its significance in immune tolerance and exploitation by cancer cells to evade immune surveillance (145–147). The blockade of this checkpoint has demonstrated potential therapeutic benefits, revealing a decrease in tumor growth and an increase in the efficacy of other immunotherapeutic strategies when the inhibitory effects of CD24 are neutralized (148).

**Figure 3:**
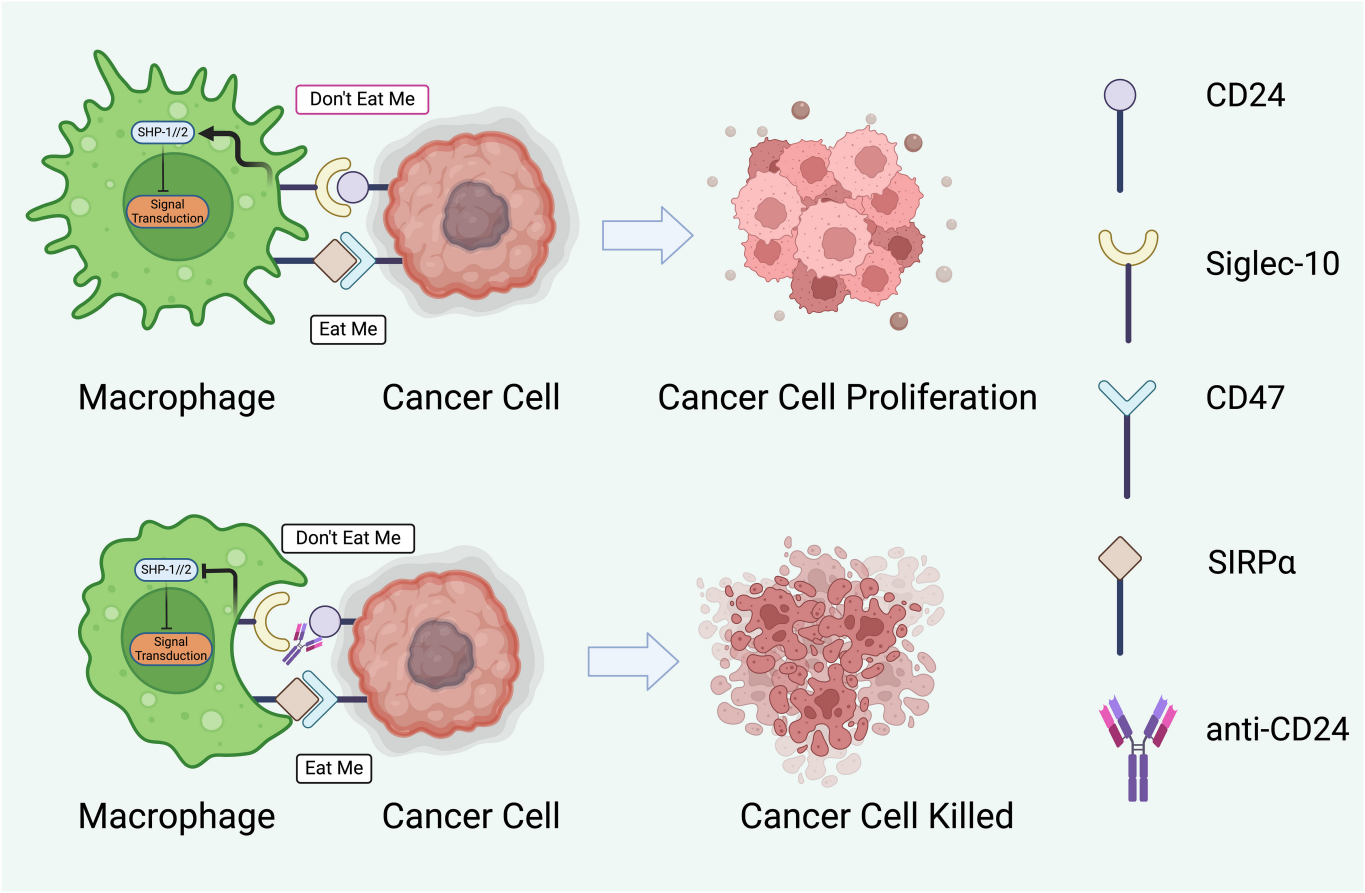
The potential therapeutic intervention to modulate interaction between macrophages and cancer cells through regulatory proteins. The cancer cell displays ‘Don’t Eat Me’ signals through proteins such as CD24, which binds to Siglec-10 on the macrophage, and CD47, which interacts with SIRPα. These interactions result in the phosphorylation of SHP-1/2 (Src homology region 2-containing protein tyrosine phosphatases) within the macrophage, leading to an inhibitory signal transduction that prevents phagocytosis. Consequently, the cancer cell evades immune destruction and proliferates (145, 150). The lower half of the figure represents a potential therapeutic strategy to subvert this immune evasion. An anti-CD24 antibody blocks the CD24- Siglec-10 interaction, thus negating the ‘Don’t Eat Me signal (151). This allows the macrophage to receive ‘Eat Me’ signals, which may be mediated by other surface molecules or opsonizing antibodies, facilitating the phagocytosis and destruction of the cancer cell. The arrow between the two sets of images indicates a therapeutic transition from unchecked cancer cell proliferation to effective immune-mediated killing. This concept represents a significant avenue of research in cancer immunotherapy, aiming to enhance the efficacy of macrophage-mediated phagocytosis as a treatment strategy. *Created with BioRender.com*.

Furthermore, the expression of CD24 and its role as a phagocytosis checkpoint varies across different tumor types and stages, suggesting a need for targeted approaches in utilizing this axis for cancer therapy (149). Investigations into the regulatory mechanisms governing CD24 expression and its interaction with Siglec-10 are critical for developing strategies to overcome this mode of immune evasion (**Figure 3**).

The exploration of CD24 as a phagocytosis checkpoint opens new avenues for cancer immunotherapy, offering insights into the intricate balance between immune activation and suppression within the tumor microenvironment. Future research focusing on the detailed mechanisms of CD24-mediated immune evasion and the development of innovative therapeutic agents targeting this pathway promises to enhance our arsenal against cancer, paving the way for more effective and precise immunotherapeutic interventions.

## CD24 and its receptors

CD24 is known to interact with a variety of cell surface receptors, including P-selectin, Siglecs, and β1 integrin, and is involved in the regulation of cell adhesion and migration (49). It has also been found to be involved in the regulation of cell differentiation and apoptosis through its interaction with the Notch signaling pathway (152).

CD24 is a cell surface protein that is expressed on a variety of cell types, including immune cells, neural cells, and cancer cells (34, 153–156). CD24 lacks intrinsic enzymatic activity but is significant due to its interactions with receptor proteins such as Siglec-10, Siglec-15, and the NKG2D receptor (56, 157–160). These interactions are essential for regulating immune responses and facilitate the immune system discern between cells of the self and those that are foreign. Additionally, the interaction with the NKG2D receptor, which is found on natural killer (NK) cells and some T cells, suggests a complex role in immune surveillance and cancer immunology (161). The binding of CD24 to these receptors can influence immune cell signaling pathways, potentially leading to immunosuppressive effects that can be exploited by cancer cells to evade immune detection and destruction (160, 162).

### CD24 and Siglecs receptor

Siglecs are proteins found on cell surfaces that interact with sialic acids (163, 164). Structurally, there are two main groups of Siglecs. The first group, comprising Siglec-1, -2, -4, and -15, has a similar structure in rodents, humans, and other vertebrates, with about 25–30% identical amino acids. The second group, related to CD33, shows a structural variation between humans and other vertebrates. However, they are highly similar to CD33, with 50–85% amino acid similarity. In humans, this group includes Siglecs from -3 to -12, -14, and -16, while in mice, it consists of Siglec-3 and Siglec-E to -H. Most Siglecs, except for a few in both humans and mice, contain motifs inside their cells that play a role in inhibitory signaling (160). The Siglec family of receptors are sialic acid-binding immunoglobulin-like lectins that are expressed on immune cells, and they have been shown to interact with CD24 in a sialic acid-dependent manner.

**Figure 4:**
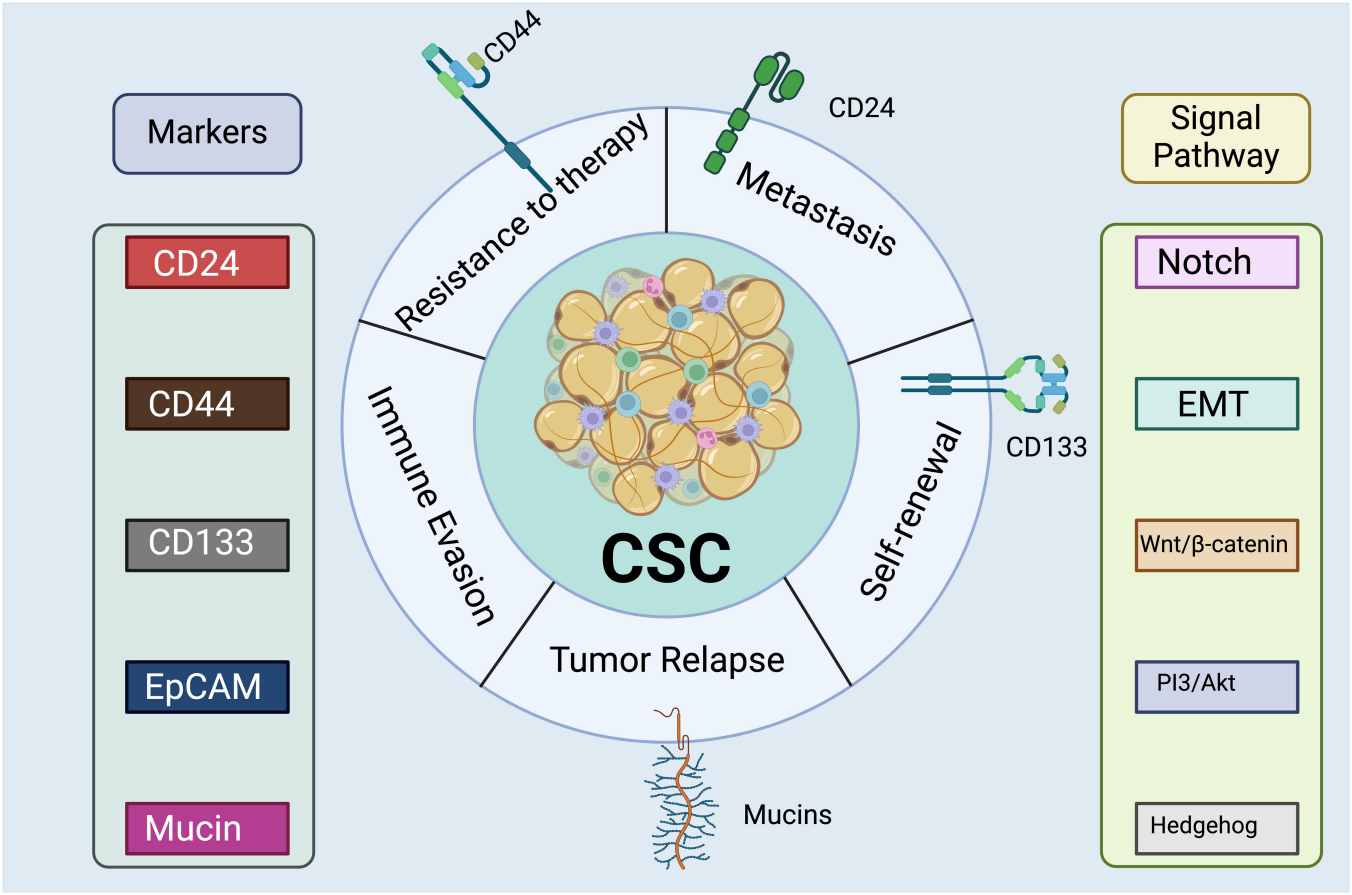
Cancer Stem Cell Markers and Signaling Pathways. The left lists surface proteins commonly expressed by CSCs, including CD24, CD44, CD133, EpCAM (Epithelial Cell Adhesion Molecule), and mucins. These markers are essential for the identification and isolation of CSCs. The right indicates intracellular signaling cascades that are activated or modulated by CSCs, such as Notch, Wnt/β-catenin, PI3/Akt, Hedgehog, and EMT (Epithelial-Mesenchymal Transition) pathway. These pathways are critical for maintaining the stemness properties of the CSCs, such as self-renewal and differentiation. There are a range of detrimental functions associated with the CSC cluster, including therapy resistance, metastasis, and tumor recurrence. The expression of CD24 has been associated with the ability of CSCs to invade and migrate, which are key steps in the metastasis of cancer cells from the primary tumor to distant organs (167). Moreover, CD24 may mediate interactions between CSCs and the extracellular matrix or other cells within the tumor microenvironment, facilitating the detachment and dissemination of CSCs (168). CD44 plays a crucial role in the resistance of CSCs to conventional therapies since it interacts with various components of the cell microenvironment, affecting cell adhesion, migration, and signaling pathways (167, 169). Recent research has discovered that CD44v3 could be used as a marker of invasive cancer stem cells driving metastasis in gastric carcinoma (170). CD133 plays a pivotal role in the self-renewal capability of CSCs which maintaining their population within the tumor (171). *Created with BioRender.com*.

Siglec-10 is a negative regulator of immune responses, and it has been shown to interact with CD24 on dendritic cells and B cells to inhibit their activation (158, 165, 166). Interaction between CD24 and Siglec-10 effectively transmits a “don’t eat me” signal, inhibiting the phagocytic activity of these immune cells against cancer cells (**Figure 4**) (145, 172). Siglec-15 is another member of the Siglec family that has been also shown to interact with CD24. It has emerged as a novel immune checkpoint molecule that negatively regulates T cell activation. Its expression on tumor cells and tumor-infiltrating myeloid cells can lead to the suppression of anti-tumor immune responses. By binding to sialic acids on the surface of T cells, Siglec-15 delivers inhibitory signals that dampen T cell activity and proliferation, thereby promoting immune tolerance to tumors (41, 159, 173–176). Siglec-E is predominantly expressed on myeloid cells, including neutrophils, macrophages, and dendritic cells (177) (178). It plays a pivotal role in the negative regulation of the innate immune response (179). The engagement of CD24 with Siglec-E on immune cells transmits inhibitory signals that modulate the immune system to respond to tumors (180). This interaction contributes to the immune evasion by downregulating the activity of phagocytic cells, thus preventing the effective clearance of tumor cells. By inhibiting the phagocytic function of immune cells, the CD24-Siglec-E axis facilitates tumor survival and progression by allowing cancer cells to escape immune surveillance (181). In addition, Siglec-E plays a crucial role in managing metabolic inflammation in obesity, which suggests the interaction between CD24 and Siglec-E in macrophages could underlie their role in attenuating metabolic inflammation and associated conditions (37, 38). However, more research is required to confirm this theory.

### CD24 and NKG2D receptor

The NKG2D receptor is an activating receptor that is expressed on natural killer cells and other immune cells (182, 183). NKG2D recognizes stress-induced ligands that are upregulated on tumor cells and infected cells (184). CD24 has been identified as a ligand for NKG2D, and this interaction has been shown to promote tumor immune evasion by suppressing NKG2D-mediated immune responses (20). The relationship between CD24 and NKG2D in the context of cancer involves a complex modulation of immune responses. While CD24 does not directly interact with NKG2D, its expression on cancer cells can influence the tumor microenvironment and the expression of NKG2D ligands on tumor cells, thereby affecting the efficacy of NKG2D-mediated immune surveillance (185).

Understanding the interplay between CD24 and NKG2D opens new avenues for cancer immunotherapy. Strategies that target CD24 expression or function in cancer cells could enhance NKG2D-mediated immune responses, potentially overcoming immune evasion mechanisms and improving the efficacy of treatments designed to activate NK and T cells against cancer (15, 167). This highlights the importance of exploring the roles of CD24 and NKG2D in cancer immunity and the potential for therapeutic interventions that modulate their activity.

In summary, CD24 is a cell surface protein that interacts with several receptor proteins, including Siglec-10, Siglec-15, and the NKG2D receptor. These interactions play important roles in immune regulation, bone homeostasis, and tumor immune evasion. Understanding the functions of CD24 and its receptors is important for the development of CD24-targeted therapies for various diseases.

## CD24-related signaling pathway

CD24 is a glycosylphosphatidylinositol-anchored protein that plays a crucial role in various physiological and pathological processes, including cancer progression. CD24-related signaling pathways are critical for mediating the functional effects of CD24 in cells (186). Here, we discuss some of the key CD24-related signaling pathways that have been identified.

### CD24-activated SRC activates cell Integrins

Integrins, a type of cell adhesion receptor, play a pivotal role in initiating intracellular signaling pathways. As a result, they are closely linked to facilitating cell attachment, invasion, and metastasis by interacting with components of the extracellular matrix (187). Baumann et al. demonstrated that CD24 has the ability to activate existing α3β1 and α4β1 integrins in rat carcinoma cell lines, such as 1AS. The activation of these α3β1 and α4β1 integrins through CD24 expression enhances tumor invasion and metastasis by encouraging cell adhesion to various extracellular components, including fibronectin, collagens I and IV, and laminin (142). Furthermore, *Runz* and colleagues characterized CD24 as a regulator of lipid rafts, serving as a control point for the activity of integrins and other proteins located within these specialized membrane microdomains (188). In specific cancer cells like A125 and MDA-MB-435S, CD24 was observed to recruit β1 integrin into lipid rafts (189). This repositioning of β1 integrin within lipid rafts influences the adhesion and migration of tumor cells (190).

CD24 enhances the mobility of tumor cells through the activation of integrin subunits (notably α3β1 and α4β1), which support tumor cell adhesion to fibronectin and various extracellular matrix elements, including collagen types I and IV, as well as laminin. This increase in tumor cell migration was evidenced in an animal tumor model by Petra.

The activation of integrins by CD24 can occur through two distinct mechanisms. First, it may involve a direct interaction between CD24 and integrins (191). However, it is important to note that co-immunoprecipitation studies did not reveal a significant association between CD24 and integrins in this context. Second, integrins may be activated by CD24-induced Src kinase. Activated Src, in turn, promotes integrin adhesion to extracellular components like fibronectin. Additionally, Src phosphorylates and stabilizes focal adhesion kinase (FAK) and paxillin, which are key players in integrin-mediated processes such as cell adhesion, migration, and metastasis (142). Consequently, CD24 appears to have a significant impact on the invasion and metastasis of tumor cells by facilitating the translocation and activation of integrins, primarily through the activation of Src kinase and potential focal interactions between CD24 and integrins.

### MAPK kinase mediated mechanisms

The MAPK (mitogen-activated protein kinase) pathway consists of three protein kinases activated in sequence (ERK, JNK, and p38 MAPK) that play vital roles in various cellular processes such as cell proliferation, differentiation, and apoptosis (192). Wang et al. demonstrated the interplay between CD24 and the MAPK pathway in colorectal cancer (CRC) (193). Their immunohistochemical analysis of human CRC tissue samples revealed that CD24 expression increases with tumor progression and is strongly associated with the expression of p-ERK1/2 and p-p38 MAPK in the tissue (193). They also showed that overexpressing CD24 in SW480 cells (human colon cancer cells) leads to their proliferation both *in vitro* and *in vivo*. This proliferation was accompanied by increased activity of Raf-1, an upstream activator of ERK1/2, and p38 MAPK. Notably, CD24 overexpression had no impact on JNK1/2 (193). The positive correlation between CD24 and MAPK expression was further confirmed through microarray analysis (194). Su et al. demonstrated that Lyn, a significant member of the Src family kinases (SFKs), plays a role in the CD24-induced activation of ERK1/2 (195). Consequently, similar to other signaling pathways, CD24-triggered Src plays a pivotal role in the activation of the MAPK pathway.

In the context of CSCs, the activation of MAPK pathways contributes to the promotion of cancer stem cell-like characteristics and the maintenance of tumorigenicity (196). Therefore, CD24 indirectly facilitates the maintenance of these cells by activating the MAPK pathways.

### HER2 mediated mechanisms

HER2, or human epidermal growth factor receptor 2, is a proto-oncogene protein that stimulates the proliferation of cancer cells. Analysis of patient specimens with breast cancer using immunohistochemistry has revealed that the expression of CD24 is more frequent (34.2%) in HER2-positive specimens compared to HER2-negative specimens (26.4%) (197). The relationship between HER2 and CD24 is intricate, and it is unclear which one influences the other upstream. Nonetheless, it appears that the link between them might involve NF-κB signaling, a pathway associated with the expression of both CD24 and HER2 (197). CD24 and HER2 contribute to the phosphorylation and activation of Akt (197). It seems that CD24’s activation of Akt is dependent on Src kinase activity, as studies have shown that lipid raft-associated Src kinases play a vital role in the activation of the PI3K-Akt signaling pathway (198).

Interestingly, it is worth noting that the Akt pathway plays a role in the regulation of NFκB (199). Consequently, CD24 and HER2, which are influenced by NFκB, seem to create a positive feedback loop in the activation of NFκB. However, further research is required to unravel the specific mechanisms underlying the association between HER2, CD24, Akt, and NFκB.

CD24 also contributes to the development of resistance to trastuzumab in HER2-expressing breast tumors through the excessive activation of Src (200). Additionally, the activation of the HER2-Akt pathway by CD24 leads to resistance to lapatinib (201). As mentioned earlier, Src may be involved in Akt activation. Thus, it appears that CD24-dependent activation of Src is associated with resistance to chemotherapy. In the context of CSCs, it is known that HER2 is connected to various signaling pathways related to stemness, including Hedgehog, Wnt, NF-kB, and JAK-STAT [as reviewed in (202)]. Although CD24 can potentially modify these pathways, the precise interplay between CD24 and HER2 must be explored within the cells to determine the role of CD24 in the activation of HER2-related signaling pathways in CSCs.

### CD24-activated SRC activates STAT3

STAT3, also known as Signal transducer and activator of transcription 3, is a cytoplasmic transcription factor responsible for regulating the transcription of genes associated with cellular responses to cytokines and growth factors. A growing body of evidence suggests that the continuous activation of STAT3 is linked to the invasion and spread of tumors. In a study by Bretz et al., it was found that CD24 has an impact on STAT3 phosphorylation and can modify the expression of genes dependent on STAT3 through the activation of Src (50). The activation of Src, in turn, enhances the STAT3 signaling pathway by phosphorylating it at tyrosine 705. Activated STAT3 forms dimers and targets genes like Cyclin D1, survivin, MMP-7, OPG, and STC-1 in cancer cells. Consequently, the role of CD24-mediated activation of STAT3 appears to be especially significant in CSCs due to its influence on genes associated with the regulation of CSCs.

Another study by Burgos-Ojeda et al. revealed that CD24-positive ovarian cancer cells notably increase STAT3 phosphorylation and express genes related to stemness, such as Nanog and c-myc, which are targets of STAT3 (203). Furthermore, STAT3 promotes the transcription of Nanog in the tumor-initiating cells of CD24-positive hepatocellular carcinoma (HCC) (204). Nanog is a transcription factor crucial for the self-renewal of stem cells. Therefore, it is evident that CD24 plays a critical role in maintaining CSCs by activating STAT3 and Nanog.

### CD24 and autophagy

Autophagy is a cellular process that involves the degradation of malfunctioning organelles and cellular components within lysosomes. Autophagy plays a significant role in maintaining cellular homeostasis and promoting cell survival (205). In the context of cancer, the role of autophagy is intricate and contentious. It can act as a tumor suppressor during cancer development or as a promoter of tumorigenesis in later stages. Numerous studies have suggested that autophagy may have a crucial role in the maintenance and invasiveness of CSCs, as reviewed in (206). However, the precise involvement of CD24 in autophagy remains unclear. Cuf et al. demonstrated that an increased expression of the CD24 gene leads to the suppression of autophagy in CD44+CD24-/low breast cancer stem-like cells (203). Their study revealed that autophagy is linked to the maintenance of these cells. It appears that the negative impact of CD24 on autophagy may be related to the activation of Akt and, subsequently, the activation of mTOR, a well-known negative regulator of autophagy (205). It is worth noting that the available data in this area are quite limited, and further research is necessary to gain a better understanding of the role of autophagy in cancer and the involvement of CD24 in this process.

### CD24-activated SRC down-regulates TFPI-2

TFPI-2, short for Tissue Factor Pathway Inhibitor 2, is a serine proteinase inhibitor of the Kunitz type. It has been identified as a gene that suppresses tumor development in various cancer types (207). TFPI-2 is secreted into the extracellular matrix and has the ability to hinder the activity of multiple matrix metalloproteases. In a study conducted by Bretz et al., an immunohistochemical analysis of primary breast cancers revealed an inverse relationship between the levels of CD24 and TFPI-2. Subsequent investigations, involving the knockdown of CD24 and Src in A125 cells, indicated that the activation of c-Src by CD24 is implicated in the reduction of TFPI-2. However, the specific signaling pathway responsible for this effect has yet to be determined (208).

Studies have demonstrated that TFPI-2 can reduce the expression of stemness markers in hepatocellular CSCs and promote the differentiation of hepatocellular carcinoma cells (209). Consequently, the downregulation of TFPI-2 due to CD24-activated Src may be a critical factor in the survival of CSCs. Hence, given the findings of these investigations and the known roles of Src kinase in cancer cells, it is suggested that Src kinase plays a pivotal role in the CD24-associated pathways in cancer and in the markers of CSCs.

### EGFR mediated mechanisms

**Figure 5:**
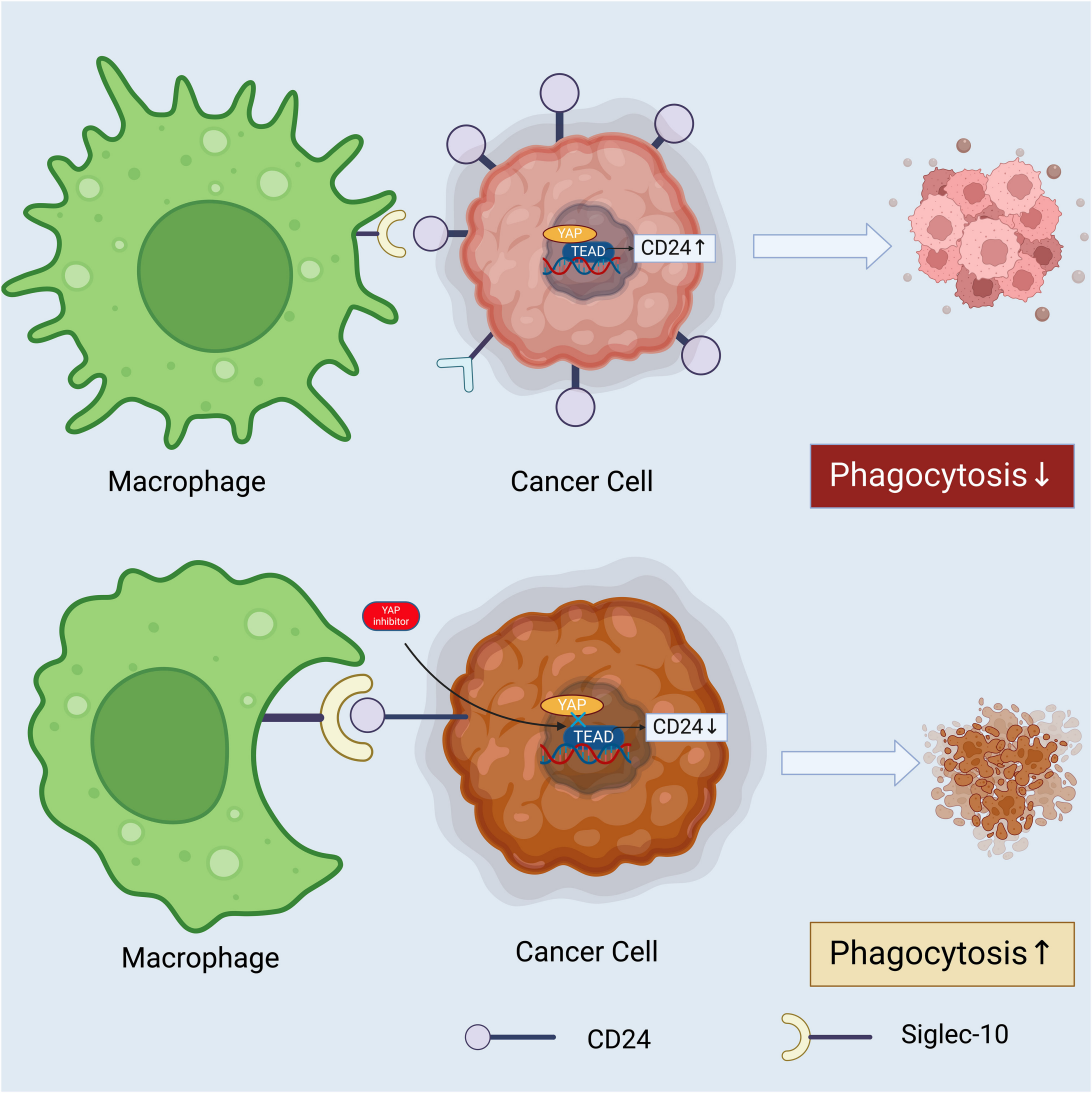
How Hippo-YAP-CD24 axis may act as a promising target to improve the prognosis of cancer patients. YAP acts a transcriptional coactivator and a core effector of the Hippo pathway, which directly activates the transcription of CD24. Elevated CD24 expression engages with the Siglec-10 receptor on a macrophage, delivering an anti-phagocytic “don’t eat me” signal, which leads to decreased phagocytosis of the cancer cell. The presence of a YAP inhibitor such as verteporfin, reduces the activity of the YAP-TEAD complex, thereby diminishing CD24 expression on the cancer cell’s surface. The decreased CD24 fails to engage the Siglec-10 receptor on the macrophage adequately, resulting in an increased phagocytic response. This underscores the potential therapeutic value of targeting the Hippo-YAP-CD24 axis to modulate immune evasion tactics employed by cancer cells (144). *Created with BioRender.com*.

EGFR, or Epidermal Growth Factor Receptor, is a cell surface signaling protein that plays a significant role in many types of human carcinomas. The relationship between CD24 expression and EGFR has been investigated through immunohistochemical analysis of gastric cancer specimens, and a positive correlation has been observed (210). Deng and colleagues further examined this correlation in gastric cancer cell lines (SGC-7901, BGC-823, and AGS-1) (210). Their research revealed that CD24 influences the expression and function of EGFR by activating RhoA, a member of the Rho family of GTPase proteins (210). Activated RhoA helps maintain EGFR expression and reduces its internalization (210). Consequently, CD24 indirectly elevates EGFR levels by limiting its movement within lipid rafts and decreasing its internalization and degradation. This reduced endocytosis of EGFR by CD24 prolongs the activation of ERK, a critical component of a signaling pathway that regulates cell proliferation, survival, and mobility (**Figure 5**).

Moreover, CD24’s positive impact on the activation of ERK1/2 and Akt (another downstream effector of EGFR) has been observed in CRC and HER2-positive breast cancer cells. ERK1/2 and Akt play roles in downregulating E-cadherin, a transmembrane glycoprotein associated with cell-cell adherens junctions. The downregulation of E-cadherin contributes to the metastasis of cancer cells (210). In this way, CD24 indirectly facilitates cancer cell metastasis by triggering the EGFR, ERK1/2, and Akt signaling pathways. Consequently, some of the unique characteristics of CSCs are partially mediated through EGFR and its downstream effectors. For instance, it has been demonstrated that the EGFR/AKT/β-catenin signaling pathway is involved in regulating CSCs in nasopharyngeal carcinoma (211). It is assumed that CD24 acts upstream in this signaling pathway because CD24 is prominently expressed in nasopharyngeal CSCs. Therefore, despite its lack of a cytoplasmic signaling domain, CD24 has the capacity to recruit crucial signaling molecules, such as EGFR, ERK, and AKT, and promote tumorigenicity in both cancer cells and CSCs.

### Wnt/β-catenin mediated mechanisms

The Wnt family consists of secreted glycolipoproteins that regulate a wide range of cellular processes by means of the transcription co-activator β-catenin. Activation of β-catenin leads to the expression of genes like Jun, Myc, and cyclin D, which are crucial for cell growth and the cell cycle (212). A study conducted by Ahmad et al. established a connection between CD24 and Wnt signaling. Their research demonstrated that CD24 engages with the Wnt pathway by activating β-catenin (213). Their immunoprecipitation experiments indicated that CD24 might have a direct interaction with β-catenin, prompting its translocation into the cell nucleus. Additionally, it has been shown that Notch and Wnt/β-catenin signaling pathways play significant roles in activating liver cancer stem cells that express CD24 (214). Consequently, CD24 can confer tumorigenic characteristics to these cells by triggering the Wnt signaling pathway.

Overall, the CD24-related signaling pathway is a complex network of molecular interactions that play important roles in regulating cell behavior in both normal and pathological conditions. CD24 is involved in the regulation of several signaling pathways that are important for cancer progression. Understanding the mechanisms by which CD24 interacts with these pathways is critical for developing effective therapies that target CD24 and its downstream signaling.

## Role of CD24 in cancer

CD24 is increasingly recognized for its multifaceted role in cancer pathogenesis, serving as a critical biomarker, prognostic indicator, and therapeutic target.

### CD24 as a cancer biomarker

CD24 has been found to be overexpressed in a variety of cancers, including B-cell lymphomas, cholangiocarcinoma, pancreatic adenocarcinoma, urothelial carcinoma, erythroleukemia, gliomas, breast cancer, small cell lung cancer, esophageal squamous cell carcinoma, hepatocellular carcinoma, ovarian cancer, primary neuroendocrine carcinomas and prostate carcinomas (8, 148, 215–229). Its overexpression has been shown to increase the adhesion of cancer cells to extracellular matrix proteins, such as laminin and collagen, and to promote cell migration and invasion.

CD24 serves as a crucial marker for both the diagnosis and prognosis of cancer. For example, the expression of CD24 on breast cancer is notably higher in invasive carcinoma compared to precancerous lesions. Moreover, the presence of CD24 on the cell surface and in the cytoplasm is associated with poor prognosis, histology grades, tumor size, and lymph node positivity (230). In non-small cell lung cancer, CD24 expression serves as an independent marker for the overall survival of cancer patients (231). Additionally, in esophageal squamous cell carcinoma, CD24 expression is also demonstrated to be linked to tumor lymph node metastasis, tumor grade, and survival time (232). Similar patterns have been observed in various other cancer types, including cholangiocarcinoma, urothelial carcinoma, ovarian cancer, and prostate carcinomas (233). Consequently, the role of CD24 as a tumor marker offers potential in diagnostic, prognostic, and therapeutic strategies, highlighting the importance of further research to exploit the role of CD24 multifaceted functions in cancer biology.

### CD24 and tumor progression

CD24 has also been shown to play a role in tumor initiation, as CD24-positive cells have been shown to have increased tumorigenic potential in animal models (234). CD24 has been implicated in tumor growth, invasion, and metastasis, and has been suggested as a potential therapeutic target for cancer treatment (235). These observations suggest a potential causative role for CD24 in cancer development, as demonstrated by experiments using small-interfering RNA to silence CD24 expression in tumor cells, leading to a direct impact on cell proliferation and survival in tissue culture (236). Antibody-blocking experiments also reveal that anti-CD24 monoclonal antibodies can inhibit the growth of human pancreatic cell lines *in vitro* (225). Significantly, targeted mutation of CD24 has been shown to reduce the size of hepatocellular carcinomas induced by the transgenic expression of hepatitis virus B genes (237). Nonetheless, further studies are necessary to fully elucidate the precise functions of CD24 in cancer pathogenesis and the underlying mechanisms at play.

### CD24 and cancer stem cells

Cancer stem cells (CSCs), also referred to as tumor-initiating cells, represent a minor fraction of tumor cells that exhibit characteristics akin to normal stem cells. This concept has gained empirical support through clinical trials, which have confirmed the link between CSCs and critical oncogenic processes such as tumor formation, metastasis, recurrence, and resistance to treatment (238). Consequently, the therapeutic targeting of CSCs has emerged as a promising strategy for the intervention and prevention of tumor progression (239–245). Currently, several potential stem cell markers are predominantly utilized for the detection and separation of CSCs from various solid tumors (239, 246–249). Among these, CD24 has been identified as a potential marker for CSCs (250). Human cancer stem cells, a subpopulation of cells within tumors that have self-renewal capacity and the ability to differentiate into various cell types, appear to have decreased expression of CD24 compared to their offspring (251). CD24-positive CSCs have been shown to be more resistant to chemotherapy and radiation therapy compared to CD24-negative cells (252). CD24 has been identified as a potential therapeutic target for CSCs, as targeting CD24 expression has been shown to reduce CSC self-renewal and sensitize cells to chemotherapy (253, 254) (**Figure 3**).

### CD24 in immune evasion

Avoidance of immune system destruction represents a fundamental characteristic of cancerous growths (255). Cancer cells are known to exploit immune checkpoint signaling pathways to transmit inhibitory signals to anti-tumor immune cells, thereby evading immune detection. There is an increasing body of evidence supporting the notion that the strategic targeting of tumor-associated macrophages to stimulate their phagocytic activity against cancer cells offers a viable therapeutic approach (256). CD47 interacts with signal regulatory protein alpha (SIRPα) to modulate the phagocytic process of macrophages (55). Crucially, recent investigations into CD47 have encountered a substantial impasse, signifying a pivotal moment in the advancement of cancer immunotherapy. The prevalent expression of CD47 on human erythrocytes and platelets presents a significant challenge, as its inhibition can lead to adverse effects including severe anemia and thrombocytopenia (150, 257). These challenges have considerably dimmed the prospects of CD47 as a viable therapeutic target, highlighting the imperative need for alternative mechanisms capable of efficiently activating the anticancer capabilities of the immune system.

**Figure 6:**
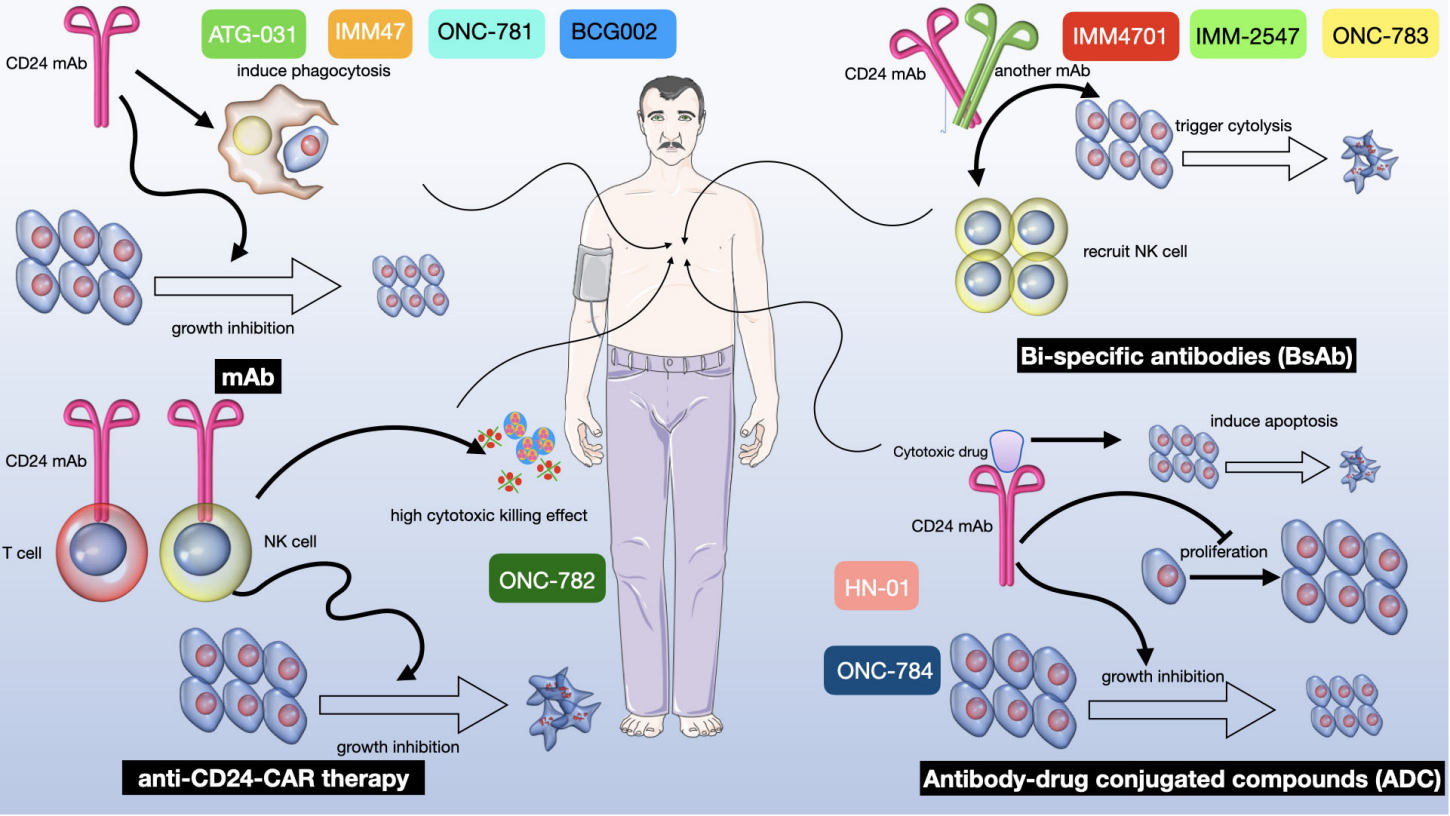
A multifaceted therapeutic approach targeting CD24 in cancer treatment. There are four distinct strategies exploiting CD24 to mediate antitumor effects: i. mAb alone: Represented by ATG-031, IMM47, ONC-781 and BCG002, CD24 mAb induces phagocytosis of tumor cells by macrophages, leading to growth inhibition of the tumor (271). ii. anti-CD24-CAR Therapy: Known as ONC-782, this therapy utilizes a CD24 mAb within a chimeric antigen receptor (CAR) on T cells to direct their cytotoxic activity towards CD24-positive tumor cells, thereby curbing tumor progression (271–273). iii. Bi-specific Antibodies (BsAb): Denoted as IMM4701, IMM-2547 and ONC-783, these antibodies are designed to bind two different antigens simultaneously. One arm of the BsAb binds to CD24 on tumor cells, while the other arm is engineered to recruit and activate NK cells, resulting in tumor cell cytolysis and subsequent growth inhibition (146, 185, 274, 275). iv. Antibody-Drug Conjugated Compounds (ADC): Labeled as “HN-01” and “ONC-784”, this therapeutic approach involves a CD24 mAb conjugated to a cytotoxic drug. The antibody targets CD24-expressing tumor cells and delivers the cytotoxic agent directly to them, inducing apoptosis and inhibiting both proliferation and tumor growth (268, 269).

CD24 has been shown to suppress the activity of natural killer cells and dendritic cells, which are important components of the immune system that play a critical role in tumor surveillance and elimination (34). CD24 has also been shown to promote the recruitment of immunosuppressive cells, such as regulatory T cells and myeloid-derived suppressor cells, to the tumor microenvironment, further contributing to immune evasion (258, 259) In addition, research led by Suzuki et al. has delineated that CD24 initiates apoptotic processes within human B cells via mechanisms associated with glycolipid-rich membrane domain (260). Further studies have extended these findings, illustrating that CD24 also facilitates apoptotic pathways in human precursor B acute lymphoblastic leukemia cells during both pro-B and pre-B phases, a process marked by the sequential activation of multiple caspases (261) (**Table 1** and **Figure 6**).

**Table 1:** Preclinical studies *in vivo* with agents that target CD24.

Tumor	Agent	Result	Reference
Breast carcinoma	scFvs	Tumor growth inhibition, increased efficacy of epirubicin	(262)
Small cell lung cancer	SWA11-SPDB- dg.ricin A chain	Tumor growth inhibition	(263–265)
Burkitt’s lymphoma	SWA11.dgA	Durable complete remissions	
Colorectal adenocarcinoma	SWA11-ZZ-PE38	Tumor growth inhibition	
Lung adenocarcinoma	SWA11	Tumor growth inhibition, increased efficacy of gemcitabine	(266)
Ovarian carcinoma	SWA11	Tumor growth inhibition	
Hepatocellular carcinoma	hG7-BM3-VcMM	Tumor growth inhibition	(267)
Hepatocellular carcinoma	G7mAb-DOX	Tumor growth inhibition, improved survival	(268)
Hepatocellular carcinoma	HN-01	Tumor growth inhibition, improved survival	(269)
Pancreatic adenocarcinoma	CAR-redirected anti-CD24 T-cells	Tumor growth inhibition, improved survival	(270)

Recent scholarly investigations have increasingly focused on the intricate relationship between the Hippo-YAP (Yes-associated protein) signaling pathway and CD24-mediated immune evasion mechanisms (144, 276). The Hippo-YAP axis, a critical regulator of cellular proliferation, apoptosis, and organ size, has been identified as a key player in the modulation of tumor microenvironments and the facilitation of cancer cell immune escape (277–279). Specifically, the interaction between the transcriptional co-activator YAP and the CD24 surface molecule delineates a novel mechanism through which cancer cells circumvent immunological destruction (144). The activation of YAP, and its subsequent effect on CD24 expression, further enhances this evasion by effectively modulating the immune system’s ability to recognize and eliminate cancer cells. The latest research by Xiumin Li et al. shows the first time how the Hippo signaling pathway modulates the phagocytic activity of macrophages through the regulation of CD24. Their findings unveil a previously unrecognized signaling continuum extending from the components of the Hippo pathway to the transcriptional activation of CD24, facilitated through the regulation of the CD24 promoter (144). This synergy between the Hippo-YAP pathway and CD24 not only underscores the complexity of tumor-immune interactions but also opens new avenues for therapeutic interventions aimed at disrupting these oncogenic signaling cascades to restore immune system competency in targeting malignancies.

### CD24 in cancer resistance

CD24 has been shown to play a role in drug resistance in cancer. Moreover, CD24 expression has also been associated with resistance to chemotherapy and targeted therapies in several cancer types, including breast, ovarian, and pancreatic cancer (280, 281).

CD24 plays a crucial role in cancer resistance through mechanisms that enhance cancer cell survival, enable immune evasion, and promote therapy resistance. It activates signaling pathways to prevent programmed cell death, allowing cancer cells to withstand chemotherapeutic and targeted therapies (281, 282). Additionally, CD24 acts as a “don’t eat me” signal, blocking the ability of immune system to recognize and destroy cancer cells, thereby facilitating tumor growth and spread (283). This protein also supports the maintenance and proliferation of cancer stem cells, contributing to tumor heterogeneity and resilience against treatments (284). Targeting CD24 aims to dismantle these protective mechanisms, potentially making cancer cells more vulnerable to treatment by enhancing immun2e response, reducing cancer stem cell renewal, and increasing the effectiveness of chemotherapy and targeted drugs.

## Clinical application of targeting CD24

CD24 has emerged as a lynchpin of tumor progression and a promising therapeutic target for anti-cancer therapy. Several clinical trials have been conducted to evaluate the safety and efficacy of CD24-targeted therapies. Here, we discuss the main progress of clinical trials of CD24 (**Figure 6**).

### Antibodies targeting CD24

There are ongoing preclinical studies and clinical trials evaluating the effectiveness of monoclonal antibodies that target CD24 in cancer therapy. For instance, CD24 has been demonstrated to be necessary for the subsequent development of lung metastases (285). Preclinical studies and clinical trials have shown that targeting CD24 can significantly reduce lung metastases in bladder cancer and target liver cancer stem cells, highlighting its role in the development of metastases and its necessity for the proliferation of certain cancer cells (286). Novel antibodies like G7 mAb have been developed to target liver cancer stem cells specifically, demonstrating the potential of CD24-targeting strategies (268, 287). Furthermore, integrating CD24 targeting with chemotherapy drugs like doxorubicin and gemcitabine has yielded encouraging outcomes in boosting the efficacy of these therapeutic approaches (8, 288).

The synergy between CD24 monoclonal antibodies and chemotherapy drugs or cytotoxic agents presents a potent strategy for combating aggressive cancers. Moreover, the development of bispecific antibodies against CD24 and other targets like CD47 and VEGF receptors introduces an additive effect on phagocytosis-mediated cancer killing, indicating a potential for improved outcomes in treatments against malignancies like glioblastoma and hepatocellular carcinoma (148, 289).

Studies indicate that blocking CD24 can boost the effectiveness of cancer treatments like sorafenib. The strategy combing CD24 inhibitors with immune checkpoint inhibitors offers a promising approach for treating aggressive cancers. For instance, elevated CD24 expression has been associated with poorer outcomes in patients, pointing to the potential of CD24 monoclonal antibodies to improve prognosis and extend life. The concept of employing bispecific antibodies (BsAbs) targeting both CD24 and PD-L1 could activate both innate and adaptive T cells immune responses, offering a more potent anti-cancer effect (290). This integrated approach underscores the need for comprehensive studies to confirm the benefits and safety of these combination treatments, including the assessment of potential drug toxicities and side effects, as well as the exploration of combining radiotherapy with CD24 targeting to stimulate a more effective tumor immune environment response.

### CAR-T cell therapy targeting CD24

Chimeric antigen receptor (CAR) T cell therapy is a promising approach for the treatment of cancer. SWA11 is a third-generation codon-optimized CAR with a highly active scFv that specifically targets the CD24 protein core. Clinical trials of SWA11 are currently ongoing, and early results have shown promising anti-tumor activity with manageable side effects (291). It has been investigated as a potential therapeutic agent for several types of cancer, including lung cancer, ovarian cancer, pancreatic cancer, triple-negative breast cancer and human colorectal cancer xenograft models (266). Moreover, SWA11 can specifically increase the antitumor efficacy in synergy with multiple chemotherapy agents, including oxaliplatin, 5-fluorouracil, irinotecan, paclitaxel, and doxorubicin (292). Another specific CAR T-cell BCMA (B cell maturation antigen)-CD24 CAR-T, a novel multiple myeloma immunotherapy, was developed by *Fumou Sun et al.* They have demonstrated strong cytotoxic activity and selectivity for multiple myeloma cells *in vitro* and *in vivo *(293).

In addition to T cells, NK cells have also exhibited the ability to kill ovarian cancer cells in laboratory studies and samples taken directly from ovarian cancer patients (294). Another approach involves the use of dendritic cells loaded with cancer cells coated with antibodies that target a variety of surface antigens, such as CD24. This technique facilitates the presentation of tumor antigens to CD8+ T cells and has been shown to increase T cell-mediated cytotoxicity in melanoma and ovarian cancer cell lines (295).

### RNA-based therapies targeting CD24

RNA interference has been proposed as a potential treatment strategy for cancer (296, 297). The use of a liposomal system to deliver CD24 shRNA (short hairpin RNA) has shown to be very promising in decreasing the expression of CD24 both *in vitro* and *in vivo *(194). Additionally, administration of shRNACD24 resulted in a significant reduction in tumor growth through inhibiting the formation of new blood vessels and promoting cell death. This indicates that gene therapy using shRNACD24 could be a new and appealing strategy for treating ovarian cancer in clinical settings (298). Data above has demonstrated that knockdown of CD24 by shRNA therapies might be a potential therapeutic approach against human ovarian cancer. However, more research is needed to fully understand the safety and efficacy of shRNACD24 therapies and to identify the optimal patient populations for these treatments.

## The progress of clinical trials for cancer therapies targeting CD24

Globally, multiple oncology clinical trials are being conducted to assess anti-CD24 therapies in preclinical settings. Herein we will review the latest studies involving anti-CD24 antibody treatments in cancer patients, comprising seven distinct investigations. In the United States, a phase 1b/II trial (NCT04060407) was designed to appraise the combined use of CD24Fc with ipilimumab and nivolumab, aiming to mitigate the toxicity associated with immunotherapy in metastatic melanoma patients who have not previously received anti-PD1/L1 inhibitors. However, this trial was discontinued for business-related reasons (145). Another American phase I/II trial (NCT04552704) sought to understand the adverse effects of CD24Fc in advanced malignant solid neoplasm and evaluate its potential to expedite recovery and lessen the severity of immunotherapy side effects. This study was also prematurely closed due to sponsor changes (151). A phase III trial (NCT04095858) intended to investigate the efficacy of CD24Fc in preventing acute graft versus host disease following myeloablative hematopoietic stem cell transplantation was halted (299). The fourth clinical trial involved 44 individuals with acute GvHD or leukemia post-Hematopoietic Stem Cell Transplantation, grouped into four cohorts for varying doses of CD24Fc. Designed as a multicenter phase IIa study, its purpose was to assess the safety and effectiveness of CD24Fc in GvHD prevention. The study was completed on May 18, 2021, with details registered under ClinicalTrials.gov Identifier NCT02663622 (39). In Israel, a study (NCT01214512) was conducted to evaluate the ability of one CD24 assay to detect colorectal adenoma via blood tests (167). Another Israeli study (NCT01265225) planned to investigate stem cell markers prognostic value for breast cancer recurrence was withdrawn due to lack of funding (145). In Egypt, a completed observational trial (NCT04907422) employed CD24-AuNC as a diagnostic marker for Carcinoma Ex Pleomorphic Adenoma of Salivary Glands, proposing CD24-AuNC as a sensitive and specific diagnostic tool (271).

These clinical efforts underscore the ongoing investigations into CD24 as a therapeutic target in various cancers, highlighting the complexities and challenges encountered in translating preclinical findings to clinical therapies.

## Conclusions

Exploring CD24 as a therapeutic target in oncology presents a unique set of advantages and challenges, reflecting its complex role in tumor biology and immune interactions. The targeting of CD24 offers significant benefits in cancer therapy due to its selective expression on tumor cells and its role in modulating immune escape mechanisms. Firstly, CD24 is often overexpressed in cancer cells, particularly in CSCs, making it a specific target for therapies that aim to reduce tumor recurrence and metastasis. This overexpression is less common in normal tissues, potentially reducing the risk of off-target effects compared to more ubiquitously expressed checkpoints. Secondly, targeting CD24 can disrupt the “don’t eat me” signal that tumor cells emit to avoid phagocytosis by macrophages. This can potentially enhance the effectiveness of the immune system in recognizing and destroying these cells. Thirdly, CD24 targeting can be combined with other forms of cancer therapy, including traditional chemotherapy, radiation, or other immunotherapies that target different pathways or mechanisms, potentially leading to synergistic effects.

Other well-known checkpoints such as CD47/SIRPα and PD-1/PD-L1 have their own sets of advantages and disadvantages. CD47 is also widely expressed on normal cells, raising concerns about potential anemia and other side effects due to the removal of healthy cells. PD-1/PD-L1 inhibitors are among the most successful immune checkpoint inhibitors used today, but they often require the presence of an existing immune response to be effective, which may not be present in all cancer patients. Activation of the CD24/Siglec-10 pathway facilitates tumor immune evasion by suppressing the activity of cytotoxic T cells and macrophages. To inhibit CD24 signaling, monoclonal antibodies, CAR T cell therapy and other methods of treatment have been exploited in preclinical studies.

Despite its potential, targeting CD24 also presents certain drawbacks, including its complex involvement in immune regulation and the potential for adverse effects due to its expression on normal tissues. Firstly, CD24 also plays roles in immune cell signaling and regulation, which are not yet fully understood. Targeting CD24 might disrupt these processes and lead to unintended immune responses or decrease immune system modulation which could be beneficial in certain contexts. Secondly, the pathways and mechanisms involving CD24 are less well-characterized compared to more studied targets like PD-1/PD-L1. This can make it harder to predict and manage potential side effects or to design effective therapies. Thirdly, expression of CD24 can vary significantly between different types of cancer and between patients. This variability can affect the efficacy of CD24-targeted therapies and may require personalized treatment approaches.

Targeting CD24 could offer a more specific approach to enhancing phagocytosis of cancer cells compared to some other checkpoints, but it also faces challenges related to its complex biology and variability in expression. Further research and clinical trials are needed to fully understand its potential and limitations. This exploration of CD24 and other immune checkpoints continues to evolve as more is learned about their roles in cancer and the immune system.

## Author contributions

KZ: Funding acquisition, Supervision, Writing – original draft. CW: Supervision, Writing – original draft. XL: Supervision, Validation, Writing – original draft. MN: Software, Writing – original draft. DW: Software, Validation, Writing – review & editing. XC: Validation, Writing – review & editing. HZ: Conceptualization, Funding acquisition, Supervision, Validation, Writing – review & editing.
